# AI-assisted tumor board decision-making in pancreatic oncology

**DOI:** 10.1186/s12911-026-03444-x

**Published:** 2026-03-20

**Authors:** Markus Mergen, Felix Busch, Benjamin Schwarberg, Pia Koldeweihe, David Jungwirth, Jonas Sydlik, H. Carlo Maurer, Marcus R. Makowski, Daniel Spitzl, Florian T. Gassert

**Affiliations:** 1 Department of Diagnostic and Interventional Radiology, School of Medicine, TUM University Hospital, Technical University of Munich, Munich, Germany; 2 Department of Surgery, TUM University Hospital, TUM School of Medicine and Health, Technical University of Munich, Munich, Bavaria Germany; 3 Medical Clinic and Polyclinic II, TUM School of Medicine and Health, TUM University Hospital, Technical University Munich (TUM), Ismaningerstr. 22, 81675 Munich, Germany

**Keywords:** Pancreatic Cancer, Large Language Models, Tumor Board, Clinical Decision-Making, Artificial Intelligence

## Abstract

**Background:**

Pancreatic cancer requires nuanced, multidisciplinary treatment planning typically conducted within tumor boards. While Large Language Models (LLMs) have shown capabilities in medical reasoning, their ability to approximate complex, integrative decision-making in oncology remains underexplored.

**Methods:**

This study evaluated the performance of LLaMA 3.3 (70b) in predicting tumor board decisions for newly diagnosed pancreatic cancer patients. Clinical documentation (including free-text imaging reports, pathology findings, and patient history) from 42 first-diagnosis cases discussed in a real-world tumor board was collected. The model was tasked with predicting one of three treatment options: surgical resection (SURG), neoadjuvant chemotherapy (NEO), or palliative therapy (PALL). Four prompting strategies were evaluated: zero-shot, advanced (adv.) zero-shot, Chain-of-Thought (CoT), and few-shot prompting. Performance was assessed using accuracy, micro- and macro-averaged F1 scores, and category-specific recall.

**Results:**

The advanced zero-shot and CoT strategies achieved the highest overall accuracy of 78.6% and a micro-averaged F1 score of 0.786. However, this performance was driven primarily by the correct classification of majority classes (SURG and PALL). Crucially, both high-accuracy strategies failed to identify any of the neoadjuvant therapy candidates (Recall NEO = 0.00; 0/7 cases), systematically misclassifying them as palliative or surgical. While few-shot prompting improved the detection of neoadjuvant cases (Recall NEO = 1.00), it introduced substantial noise, reducing overall accuracy to 56.7%. LLaMA 3.3 (70b) demonstrates high concordance with tumor board decisions for clear-cut surgical or palliative cases but exhibits a critical systematic failure in identifying candidates for neoadjuvant therapy. The high global accuracy masks a significant safety limitation regarding the recognition of complex, intermediate-stage patients.

**Conclusion:**

These findings suggest that current LLMs may approximate majority-class decisions but risk overlooking curative treatment pathways in nuanced scenarios, necessitating rigorous oversight and specific adaptation before clinical consideration

**Supplementary Information:**

The online version contains supplementary material available at 10.1186/s12911-026-03444-x.

## Introduction

Pancreatic ductal adenocarcinoma (PDAC) remains among the most aggressive and lethal solid tumors, with a five-year survival rate of around 10% [1–3]. Due to its often late presentation and complex anatomical and biological behavior, treatment planning for pancreatic cancer requires careful integration of multimodal diagnostic information and expert judgment [4–7]. Consequently, multidisciplinary tumor boards are the standard for therapeutic decision-making in these patients [8–10]. These boards synthesize data from various sources, including cross-sectional imaging (CT, MRI), interventional diagnostics (endoscopic ultrasound, ERCP), pathology, and clinical history, to reach individualized treatment recommendations [8, 11–13].

The decisions made within these multidisciplinary tumor boards are critically important, as they directly impact patient outcomes. Multiple studies have demonstrated that such integrated, collaborative approaches lead to improved treatment efficacy, more accurate staging, and ultimately better survival rates for patients with pancreatic cancer [8, 14, 15]. This underscores the essential need for comprehensive evaluation of all relevant clinical and diagnostic modalities by experts from various disciplines to ensure optimal therapeutic strategies.

In recent years, large language models (LLMs) have shown remarkable capabilities in processing unstructured text and mimicking domain-specific reasoning tasks, including those in the medical field [16, 17]. While prior studies have explored the application of LLMs in medical summarization, question answering, and guideline retrieval, their potential to support or replicate complex, integrative clinical decision-making as seen in pancreatic tumor boards still remains relatively unexplored [18–21].

Therefore, this study investigates whether LLaMA 3.3 (70b), a state-of-the-art open-weight LLM, can approximate real-world tumor board decisions in newly diagnosed pancreatic cancer cases.

## Materials and methods

This retrospective study was approved by the Institutional Review Board of the Technical University of Munich (Approval ID: 2024-590-S-CB) and conducted in accordance with all applicable ethical and data protection regulations. The requirement for individual informed consent was waived by the ethics committee due to the retrospective design and the use of fully anonymized clinical data.

The study focused exclusively on first-diagnosis cases. The ground truth reference was formed by the actual tumor board decisions, which assigned each patient to one of three predefined treatment options: surgical resection (SURG), neoadjuvant chemotherapy (NEO), or palliative therapy (PALL).

Treatment allocation followed the NCCN Clinical Practice Guidelines in Oncology for Pancreatic Adenocarcinoma. Patients assigned to the Surgical Resection (SURG) group were classified as “Resectable” according to NCCN criteria, defined by the absence of arterial tumor contact and no unreconstructable venous involvement, provided they were medically fit for surgery. The Neoadjuvant Therapy (NEO) category was defined according to NCCN and S3 guidelines, encompassing not only anatomical borderline resectability (Type A) but also high-risk biological features (Type B) and specific patient conditions (Type C). Specifically, ‘Borderline Type B’ cases included patients with technically resectable or borderline tumors but high biological risk, such as CA 19 − 9 levels > 500 U/ml (without significant cholestasis) or suspicious regional lymphadenopathy. While ‘induction chemotherapy’ is the standard clinical terminology for patients with locally advanced pancreatic cancer (LAPC), these cases were included in the NEO category for this study as they share a curative-intent pathway aimed at potential downstaging and secondary resection. Finally, Palliative Therapy (PALL) was assigned to patients with proven distant metastases or locally advanced disease deemed permanently unresectable due to extensive vascular involvement or poor performance status, where the treatment intent was life-prolonging or symptom-controlling rather than curative. In a few cases, patients with oligmetastatic PDAC in the HOLIPANC/METPANC study were treated in a neoadjuvant setting. In cases of equivocal findings, the final consensus of the multidisciplinary board served as the definitive label.

The tumor board consisted of specialists from surgery, medical oncology, radiology, pathology, and gastroenterology. Regular meetings were held to discuss each case in detail and to reach consensus-based treatment recommendations. Tumor board decision was regarded as the gold standard for treatment decision.

We evaluated the offline performance of the open weight large language model LLaMA 3.3 (70b), deployed securely within institutional infrastructure to ensure compliance with data privacy and security standards. Fully anonymized clinical texts were concatenated in a standardized order (1. Clinical History, 2. Imaging Reports, 3. Pathology Findings) and separated by clear, standardized headers. Only patients with complete clinical documentation (imaging, pathology, and history) were included. To prevent ‘label leakage,’ we removed not only the final tumor board decisions but also any language referring to already initiated or planned downstream treatments (e.g., ‘patient scheduled for resection on…’…).

No model fine-tuning was performed. Instead, four prompting strategies were systematically tested to guide the model’s treatment recommendation:

- Zero-shot prompting (temperature = 0.0, top_p = 0.9).
- Adv. zero-shot prompting (temperature = 0.0, top_p = 0.9).
- Chain-of-thought + self-consistency prompting; for every report, *N* = 7 independent reasoning traces were sampled (temperature = 0.7, top_p = 0.95). The majority label after stripping the final line was taken as the prediction.
- Few-shot prompting with 4 examples per class (few-shot); from a total of 42 cases, 4 representative cases were selected as examples for each prompting strategy, and the remaining cases were used for evaluation.

Prompts can be found in Suppl. Table 1.

Prompting templates constrained the model to select exactly one of the three treatment categories: SURG, NEO or PALL.

Performance was assessed using accuracy, precision, recall, micro and macro-averaged F1 scores across the treatment categories. The F1 score was calculated as the harmonic mean of precision (also known as positive predictive value) and recall (also known as sensitivity). The micro scores were computed by aggregating the true-positive, false-negative, and false-positive findings across all classes. The macro scores were computed by calculating the scores for each class individually and then averaging them, giving equal weight to each class regardless of its size.

Additionally, a category-specific error analysis was conducted to investigate failure patterns depending on the recommended therapy. All analyses were performed using Python (NumPy 1.26.4, pandas 2.2.0, scikit-learn 1.4.0, statsmodels 0.14.1, matplotlib 3.8.2, seaborn 0.13.2) [22–26]. We estimated the 95% CI case-level bootstrap resampling with 10,000 iterations. For the NEO Recall across all four prompting strategies, the Clopper-Pearson (exact) method was used, which is the standard for small sample sizes and extreme proportions (0 or 1).

## Results

**Fig. 1:**
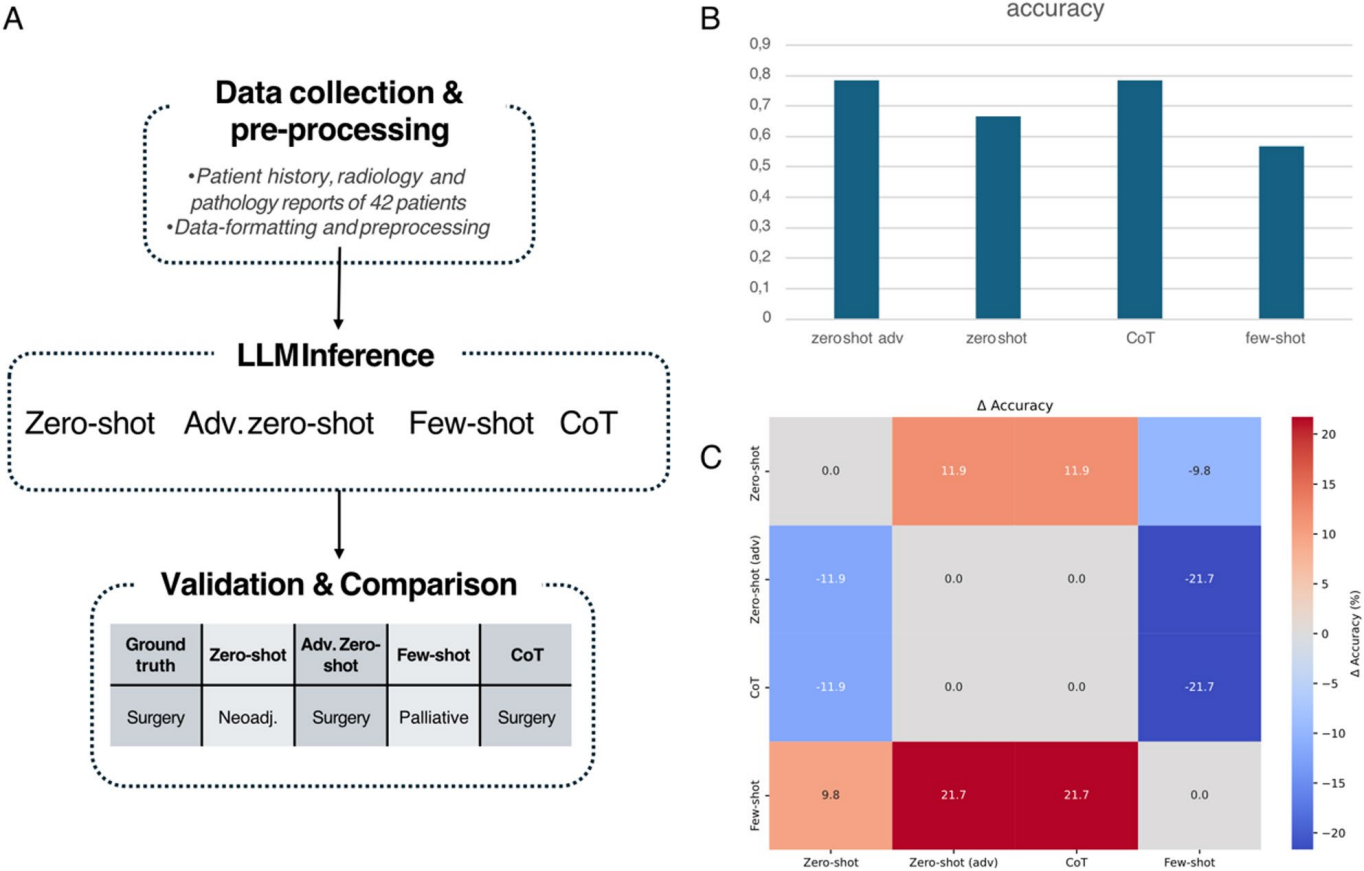
(**A**) Schematic overview of the workflow for the board approximation. After data acquisition and data pre-processing, LLaMA 3.3 (70b) was prompted in a zero-, adv. zero-, few-shot and CoT manner to predict therapy classification. (**B**) Bar chart illustrating the accuracy scores for correct therapy classification. (**C**) Heatmap depicting pairwise accuracy differences for each prompting strategy used. Positive (red) cells indicate a higher performance for the first named compared to the second named strategy, while negative (blue) cells indicate lower performance

We evaluated four prompting strategies—zero-shot, adv. zero-shot (a prompt-engineered variant with explicit task structure and label definitions), few-shot, and CoT —for predicting the correct tumor board decision among three classes: SURG, NEO and PALL. Zero-shot, adv. zero-shot, and CoT were assessed on 42 cases, whereas the few-shot setting relied on 30 cases because support examples had to be held out. The experimental workflow can be seen in Fig. 1A. Study population baseline characteristics can be seen in Table 1.

**Table 1:** Patient demographics and clinical characteristics

Characteristic	Total (*N* = 42)	SURG (*n* = 12)	NEO (*n* = 7)	PALL (*n* = 23)
**Demographics**				
Age, median (range)	66.0 (40–85)	68.5 (53–82)	63.0 (50–68)	68.0 (40–85)
Sex				
Female	21 (50.0%)	4 (33.3%)	6 (85.7%)	11 (47.8%)
Male	21 (50.0%)	8 (66.7%)	1 (14.3%)	12 (52.2%)
**Tumor Location**				
Head (Caput)	14 (33.3%)	6 (50.0%)	2 (28.6%)	6 (26.1%)
Body (Corpus)	2 (4.8%)	0 (0.0%)	1 (14.3%)	1 (4.3%)
Tail (Cauda)	4 (9.5%)	0 (0.0%)	1 (14.3%)	3 (13.0%)
Combined / Overlapping	22 (52.4%)	6 (50.0%)	3 (42.9%)	13 (56.5%)
**Vascular Involvement**				
**Arterial Contact**				
SMA (A. mesenterica sup.)	7 (16.7%)	1 (8.3%)	**2 (28.6%)**	4 (17.4%)
Celiac Axis (Tr. coeliacus)	5 (11.9%)	0 (0.0%)	**1 (14.3%)**	4 (17.4%)
Common Hepatic Artery	3 (7.1%)	0 (0.0%)	1 (14.3%)	2 (8.7%)
**Venous Contact**				
Portal Vein (PV)	7 (16.7%)	0 (0.0%)	**2 (28.6%)**	5 (21.7%)
SMV (V. mesenterica sup.)	7 (16.7%)	1 (8.3%)	**2 (28.6%)**	4 (17.4%)
**Metastasis**				
Liver Metastasis	17 (40.5%)	0 (0.0%)	3 (42.9%)	14 (60.9%)
Peritoneal / Ascites	5 (11.9%)	0 (0.0%)	0 (0.0%)	5 (21.7%)
Lung	2 (4.8%)	0 (0.0%)	0 (0.0%)	2 (8.7%)

Baseline characteristics of the study cohort (*n* = 42), stratified by the ground-truth treatment decision determined by the multidisciplinary tumor board. Data are presented as median (range) for continuous variables and count (percentage) for categorical variables. Abbreviations: SURG = Surgical Resection, NEO = Neoadjuvant Therapy, PALL = Palliative Therapy, SMA = Superior Mesenteric Artery, SMV = Superior Mesenteric Vein

adv. zero-shot and CoT achieved the highest overall performance, each reaching an accuracy and micro-averaged F1 of 0.786. Both approaches outperformed the basic zero-shot setting, which attained an accuracy of 0.667, corresponding to an absolute gain of 11.9% points. The few-shot configuration yielded the lowest overall accuracy (0.567) (Fig. 1B). Pairwise performance differences in accuracy can be seen in Fig. 1C. Statistical comparison of prompting strategies can be seen in Table 2.

**Table 2:** Statistical comparison of prompting strategies

Comparison	Accuracy Model 1	Accuracy Model 2	*p*-value	Significance
Zero-shot vs. Adv. Zero-shot	73.3%	86.7%	0.219	n.s.
Zero-shot vs. CoT	73.3%	86.7%	0.219	n.s.
Zero-shot vs. Few-shot	73.3%	56.7%	0.227	n.s.
Adv. Zero-shot vs. CoT	86.7%	86.7%	1.000	n.s.
Adv. Zero-shot vs. Few-shot	86.7%	56.7%	**0.035**	significant
CoT vs. Few-shot	86.7%	56.7%	**0.035**	significant

Pairwise comparison of overall accuracy between the four prompting strategies using McNemar’s test for paired nominal data for the 30 cases which underwent all four prompting strategies. Note: Accuracy values in this table differ from global metrics because they are calculated solely on the subset of 30 cases used for pairwise comparison with the few-shot strategy

### Class-wise performance

Class-wise analysis revealed distinct behaviors for the three decision categories. In the zero-shot condition, SURG was recovered without omission (recall = 1.000, F1 = 0.667) at the cost of moderate precision (0.500). NEO exhibited very high precision (1.000) but poor recall (0.143), showing that the model almost never selected this label. PALL was classified robustly (F1 = 0.750). Under adv. zero-shot prompting, SURG improved across metrics (F1 = 0.846) and PALL remained strong (F1 = 0.863), but NEO collapsed entirely (precision = recall = 0.000), indicating a systematic avoidance of this minority class. The few-shot strategy inverted this issue: NEO recall rose to 1.000 while precision fell to 0.200, whereas SURG recall dropped to 0.250 (F1 = 0.364), and PALL maintained perfect precision with a moderate recall of 0.632. CoT mirrored the adv. zero-shot pattern, again delivering strong results for SURG and PALL (F1 = 0.846 and 0.863, respectively) but failing to capture any NEO instances. These findings demonstrate that prompt design can redistribute errors among classes without necessarily resolving the core difficulty of predicting the NEO label. Results can be seen in Table 3.

**Table 3:** Detailed classification performance across prompting strategies

Prompting Strategy	Class	Precision	Recall	F1-Score	*n*
Zero-shot	SURG	0.500	1.000	0.667	12
	NEO	1.000	0.143 (0.004– 0.579)	0.250	7
	PALL	0.882	0.652	0.750	23
	*Macro Average*	*0.794 (0.524–0.810)*	*0.598* (*0.397–0.870)*	*0.556 (0.500–0.727)*	*42*
	Overall Accuracy			0.667 (0.524–0.810)	42
Adv. Zero-shot	SURG	0.786	0.917	0.846	12
	NEO	0.000	0.000 (0.000- 0.410)	0.000	7
	PALL	0.786	0.957	0.863	23
	*Macro Average*	*0.524 (0.428–0.607)*	*0.624 (0.553–0.667)*	*0.570 (0.488–0.629)*	*42*
	Overall Accuracy			0.786 (0.643–0.905)	42
Few-shot	SURG	0.667	0.250	0.364	8
	NEO	0.200	1.000 (0.292-1.000)	0.333	3
	PALL	1.000	0.632	0.774	19
	*Macro Average*	*0.622 (0.400–0.733)*	*0.627 (0.370–0.786)*	*0.490 (0.302–0.763)*	*30*
	Overall Accuracy			0.567 (0.400–0.733)	*30
Chain-of-Thought (CoT)	SURG	0.786	0.917	0.846	12
	NEO	0.000	0.000 (0.000-0.410)	0.000	7
	PALL	0.786	0.957	0.863	23
	*Macro Average*	*0.524 (0.643–0.905*	*0.624 (0.428–0.607)*	*0.570 (0.553–0.667)*	*42*
	Overall Accuracy			0.786 (0.643–0.905)	42

Class-wise precision, recall, and F1-scores for Surgical (SURG), Neoadjuvant (NEO), and Palliative (PALL) categories. 95% CI in brackets. Note the systematic trade-off between overall accuracy (driven by SURG/PALL) and the identification of the minority NEO class. While Adv. Zero-shot and CoT maximize overall metrics, they fail to identify NEO cases (Recall = 0.00). Few-shot prompting improves NEO recall but drastically reduces precision and overall accuracy

### Confusion matrix analysis

**Fig. 2:**
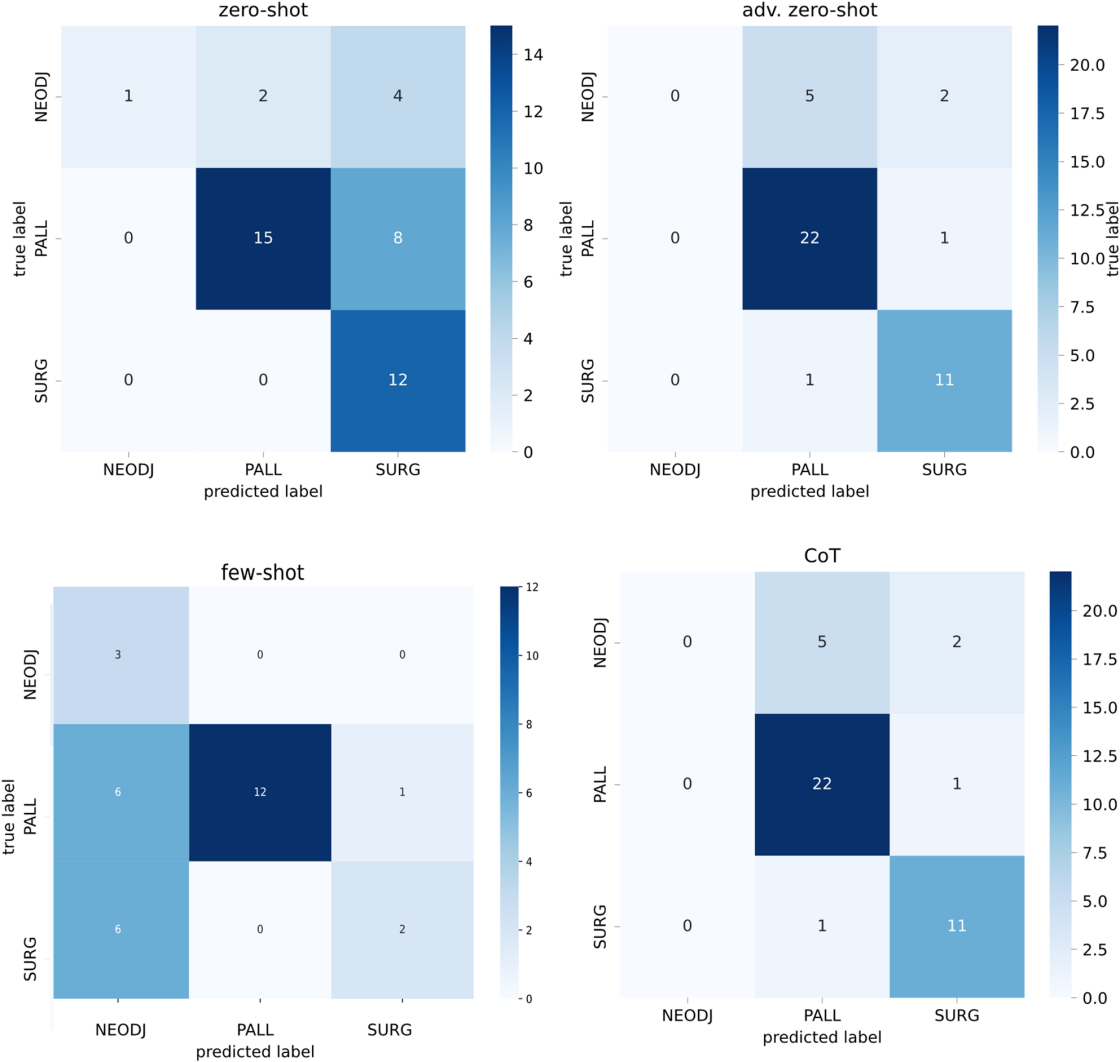
Confusion matrices comparing the multi-class classification performance of four prompting approaches—zero-shot, adv. zero-shot, few-shot and CoT. Each matrix plots the true labels on the y-axis against the predicted labels on the x-axis, with color intensity corresponding to the number of instances in each cell (darker shades indicate higher counts)

Figure 2 summarizes the misclassification patterns for the four prompting strategies. In the basic zero-shot condition, errors concentrated almost exclusively on the minority class. Six of the seven NEO cases were reassigned to other labels—four to SURG and two to PALL—resulting in the very low recall observed for this class. PALL was also frequently confused with SURG (eight of 23 cases), whereas SURG itself was recovered without omission, consistent with its perfect recall in the summary metrics.

The adv. zero-shot prompt markedly reduced noise for the majority classes but effectively suppressed NEO altogether. None of the NEO cases were correctly identified; instead, five were mapped to PALL and two to SURG. PALL and SURG, by contrast, were classified with high fidelity (only one SURG case shifted to PALL), explaining the improvement in global accuracy despite the persistent blind spot for NEO.

The few-shot model inverted this error profile. Providing exemplars encouraged the model to predict NEO, yielding perfect recall for that class, but at the expense of precision: many SURG and PALL reports were incorrectly redirected to NEO (six SURG and six PALL errors into the NEO column), which drove the sharp drop in SURG recall and overall accuracy. PALL maintained perfect precision—no samples predicted as PALL were wrong—but still suffered from substantial leakage into NEO and SURG.

Finally, the CoT strategy reproduced the pattern seen with adv. zero-shot. NEO remained unrecognized (all seven cases diverted to PALL or SURG), while SURG and PALL were classified with high consistency (only a single SURG case mislabeled as PALL). The CoT reasoning therefore stabilized majority-class decisions but did not resolve the systematic failure on the minority NEO label.

Taken together, the matrices show that prompt design mainly shifts where the inevitable errors occur: strategies that maximize overall accuracy (adv. zero-shot and CoT) do so by avoiding uncertain NEO predictions, whereas the few-shot approach overcorrects and introduces large-scale misassignment into NEO.

### Detailed error analysis

To interrogate the nature of misclassifications, every discordant case, predicted by our best prompting strategy (adv. zero-shot), was reviewed in depth. For each instance, we extracted the tumor board’s final recommendation together with the documented clinical rationale, and we asked the language model to explain why it chose its own treatment strategy and why it did not select the board’s plan. The resulting justifications were qualitatively coded and compared with the human rationale, which allowed us to identify recurrent reasoning failures rather than treating errors as isolated mislabels.

Across the discrepant cases several consistent patterns emerged. The model frequently misjudged the boundary between neoadjuvant intent and palliation. When extensive vascular involvement was present but distant metastases were absent or equivocal, it tended to default to a PALL decision and overlooked the board’s use of NEO therapy as a bridge to potential resection. Conversely, in the few situations where the model favored immediate SURG, it relied on a simplistic notion of technical resectability based solely on limited vessel contact, whereas the board weighed the likelihood of achieving negative margins and the feasibility of vascular reconstruction. Indeterminate extra-pancreatic lesions were often overinterpreted as definitive metastases, prompting premature PALL recommendations. In one instance the model failed to commit to any of the predefined strategies and offered a diagnostic rather than therapeutic answer, indicating difficulty handling uncertainty within a forced-choice framework.

Taken together, these patterns show that the model’s internal heuristics diverge most strongly from expert reasoning at three decision junctures: interpreting vascular encasement in relation to resectability, valuing equivocal metastatic findings, and recognizing NEO therapy as an active curative pathway rather than merely a fallback when SURG is impossible. The qualitative analysis therefore highlights concrete clinical rules—especially around vascular reconstruction thresholds and the management of indeterminate metastases—that need to be encoded or explicitly prompted if LLMs are to align reliably with tumor board decisions. Table 4 summarizes the human and model rationales side by side.

**Table 4:** Detailed error analysis

Case	Board plan	Rationale for board plan	Predicted strategy	Rationale for predicted strategy	Arguments against board Plan
1	NEO	• 3.5 × 2.2 cm tumor in the pancreatic body • Arterial infiltration • Superior mesenteric artery (SMA) encasement < 180° over 3 cm • Coeliac trunk involvement ≤ 180° over 1 cm • Contact with the superior mesenteric vein ≤ 180°, no portal vein invasion • No definite distant metastases (splenic and pulmonary lesions indeterminate)	PALL	• Locally advanced pancreatic cancer with encasement of the SMA • Infiltration of adjacent structures (spleen) • Indeterminate hypodense splenic lesions—possible metastases • Solitary pulmonary nodule—possible metastasis	• No clear evidence for a curative resection • Locally advanced tumor growth • Infiltration of adjacent structures
2	NEO	Tumor: 3.9 × 3.8 cm pancreatic head carcinoma Arteries: SMA contact ≤ 180° (1.2 cm), no TC/AHC infiltration Veins: SMV contact > 180° (3.5 cm), portal vein free Distant spread: none (liver clear, 4 mm lung nodule likely benign)	SURG	• Resectability: tumor located in the pancreatic head, size ~ 3–4 cm allows resection • Arterial control: contact with the SMA and aorta is limited, permitting surgical intervention • No distant metastases: increases likelihood of curative resection	• Tumor size/location: relatively large and in contact with key vessels—neoadjuvant therapy not strictly mandatory • No signs of diffuse tumor spread that would necessitate neoadjuvant treatment • Surgical resectability is present, allowing primary surgery
3	SURG	Primary tumor: 4 cm adenocarcinoma in the uncinate process Metastases: two lesions ≤ 15 mm in liver segment VI, diffusion-restricted / contrast-enhanced—metastasis suspected Vascular relation: no relevant arterial infiltration; no portal vein or lung metastases	PALL	• Presence of liver metastases (two lesions in segment VI) • Primary pancreatic ductal adenocarcinoma ~ 4 cm → Palliative therapy often best option in metastatic disease to improve quality of life and control symptoms	• Liver metastases worsen prognosis and reduce the chance of successful surgery • Tumor size and location may complicate surgery • Symptom burden and weight loss indicate advanced disease—supports palliative approach
4	NEO	Primary tumor: 2.6 × 1.9 cm adenocarcinoma of the pancreatic head Vascular relation: 360° encasement of the coeliac trunk, common hepatic artery and splenic artery; 180° SMA contact; 360° encasement of portal vein and SMV (lumen narrowed to 4 mm) Distant disease: none in solid organs	PALL	• Locally advanced pancreatic cancer with encasement of major vessels (coeliac trunk, hepatic and splenic arteries, portal vein, SMV) • Metastasis-suspect lymph nodes • No prospect of an R0 resection	• No evidence for curative resection after neoadjuvant therapy • Extensive local tumor growth with vascular invasion • Metastasis-suspect lymph nodes argue against a curative concept
5	NEO	4.5 cm tumor near pancreatic head/body junction with SMV/portal vein occlusion, long segment SMA (< 180°) and common hepatic artery contact	—	• High suspicion of pancreatic cancer • Large pancreatic head tumor with vascular infiltration (SMA, SMV, splenic artery and vein) • Contact with the left renal vein and a mass at the left adrenal gland carcinoma	• Extent of tumor and infiltrations remains uncertain • Need for accurate diagnosis and staging before neoadjuvant therapy
6	PALL	Morphology: 5.7 × 3.8 cm cystic–solid tumor in the pancreatic head Vascular contact: >180° of the SMV (non-reconstructable); no relevant arterial involvement Distant spread: CT thorax / MRI abdomen without metastases or nodal disease	SURG	• Localized tumor growth without distant metastases • No invasion of critical vessels (superior mesenteric vein only partially encased) • Potential R0 resection achievable by surgical intervention	• No evidence of an advanced, inoperable malignancy • The patient has not yet developed severe symptoms necessitating palliative therapy • Surgical resection still offers a curative option; purely palliative measures would be premature
7	NEO	Primary tumor: 4.6 × 5.9 cm pancreatic body carcinoma Vascular contact: >180° encasement of the coeliac trunk & common hepatic artery; >180° portal/SMV contact; ≤180° SMA contact Metastases: no	PALL	• Locally advanced pancreatic cancer infiltrating the coeliac trunk and confluence of the portal system • Irresectable due to tumor size and location	• Tumor not resectable due to coeliac trunk and confluence infiltration • No possibility of an R0 resection • Surgery would not improve survival and could worsen quality of life
8	NEO	Tumor: 5.9 × 4.6 cm pancreatic body/tail carcinoma Vascular relation: 360° encasement of the coeliac trunk (without aortic involvement), > 180° contact of the common hepatic artery & portal/SMV trunk, ≤ 180° SMA; splenic vein occluded, splenorenal shunt	PALL	• Locally advanced tumor size and extent • Infiltration of multiple major vessels (coeliac trunk, SMA, splenic vein) • Multivisceral involvement (pancreas, liver, spleen, left adrenal gland) • No possibility of an R0 resection	• Extensive local disease with major vessel infiltration • Multivisceral involvement and nodal metastases • No possibility of R0 resection; high operative mortality • Palliative therapy prioritises symptom control and quality of life
9	NEO	Tumor: ~3 cm pancreatic head carcinoma Vascular contact: obliteration of the portal venous confluence with collaterals (≥ 180° PV/SMV), long-segment SMA contact (> 180°), coeliac trunk & hepatic artery free Distant spread: no organ or nodal metastases	SURG	• Localised pancreatic cancer without hepatic metastasis • No suspicious locoregional lymphadenopathy • Tumor size 3 cm, potentially resectable • Cholestasis due to tumor infiltration—operative drainage feasible	• No evidence of advanced disease requiring neoadjuvant therapy • Tumor is potentially resectable—primary surgery is possible • Neoadjuvant therapy would delay operative management

Shown are falsely classified cases by the adv. zero-shot prompting strategy with the original board plan, the main reason(s) for their decision, the LLM’s prediction with its explanation for its decision as well as against the board’s decision

## Discussion

This study evaluates the capability of large language models such as LLaMA 3.3 (70b) to interpret complex, multimodal clinical documentation to support pancreatic cancer tumor board decision-making. By evaluating four prompting strategies—zero-shot, adv. zero-shot, few-shot, and CoT—we observed that prompt design substantially impacts model performance. Both adv. zero-shot and CoT achieved the highest accuracy and micro-averaged F1 score of 0.786, outperforming the basic zero-shot approach by an absolute margin of 11.9% points. These findings highlight the critical role of explicit task structure and reasoning scaffolds in eliciting reliable clinical predictions from LLMs.

Notably, the lowest performance was observed in the few-shot setting, likely due to the limited case pool available after support examples were withheld. The consistently strong performance of CoT and adv. zero-shot prompts across accuracy, precision, recall, and F1 metrics suggests that thoughtfully engineered prompts can meaningfully enhance the model’s alignment with expert consensus. However, while Chain-of-Thought reasoning can stabilize model predictions, the potential for ‘black-box’ reasoning errors necessitates a shift toward evidence-based, guideline-cited rationales to ensure clinical safety and transparency in tumor board decision support.

However, from a tumor-board perspective, under-calling NEO risks forgoing a curative pathway, whereas over-calling NEO could delay indicated surgery or palliation. Our results indicate that “high accuracy” setups (advanced zero-shot, CoT) achieve their gains partly by avoiding NEO decisions; few-shot takes the opposite trade-off. Reporting both macro/micro metrics and the class-specific confusion patterns is therefore essential for clinical interpretation.

The selection of three clinically relevant treatment categories, surgical resection, neoadjuvant chemotherapy, and palliative chemotherapy, allowed for a targeted evaluation aligned with real-world therapeutic pathways. This design not only reflects current standards of pancreatic cancer management but also highlights the model’s capability to navigate nuanced clinical considerations embedded in free-text imaging, pathology, and anamnesis reports. Such interpretive ability is essential for any AI system aspiring to support or augment multidisciplinary tumor boards, where heterogeneous and often unstructured data must be synthesized rapidly to inform critical decisions.

Importantly, this investigation leveraged authentic, de-identified clinical documentation and actual tumor board decisions, thereby enhancing the ecological validity of the findings. This real-world basis distinguishes our approach from many prior studies relying on curated or simulated data and reinforces the potential translational relevance of LLMs in oncology practice. Nonetheless, limitations remain: the relatively small cohort of 42 patients restricts statistical power and generalizability. Crucially, performance estimates for the NEO category—especially in the few-shot setting with only n = 3 evaluation cases—are statistically unstable. The observed few-shot Recall of 1.00 for NEO should therefore not be interpreted as a robust minority-class correction, but rather viewed with caution given the sample size constraints. The retrospective single-center study design precludes assessment of how model recommendations might influence clinical outcomes or workflow integration in a prospective setting. While the board’s consensus served as a “gold standard” for this study, it does not represent an absolute, immutable truth. Furthermore, the forced-choice prompting framework may induce errors by compelling the model to select a definitive treatment even in cases characterized by high clinical uncertainty. Furthermore, this design utilized in this study represents a significant simplification of clinical reality. Future iterations of LLM-based decision support should incorporate formal abstention criteria or ‘confidence-based deferral’ mechanisms, ensuring that cases characterized by high clinical ambiguity—such as indeterminate metastatic findings—are explicitly flagged for prioritized multidisciplinary review rather than forced into rigid treatment categoriesThe failure of the model in specific cases (e.g., Case 3, Table 4) highlights that simple heuristics, such as equating suspected metastasis with palliative intent, are insufficient to capture the nuanced, protocol-driven decisions of a multidisciplinary board. This suggests that while traditional rules-based systems might achieve baseline accuracy, they lack the interpretive capacity required for complex pancreatic cancer management.

Any future clinical application would require prospective validation, larger multi-center datasets, and a shift toward evidence-based, guideline-cited rationales to ensure safety and transparency.

Currently existing literature has begun to explore the application of large language models in healthcare, particularly for natural language understanding tasks such as summarization, report generation, and information extraction [27–29]. However, much of this research has focused on general clinical documentation or narrow diagnostic tasks rather than complex, multimodal decision-making scenarios like tumor board deliberations [30, 31]. Studies utilizing models like GPT-4 and Med-PaLM have demonstrated promising performance in question answering and clinical reasoning tasks but often rely on synthetic or benchmark datasets [32, 33]. Few investigations have tackled high-stakes, real-world oncology use cases where diverse data sources, including pathology, radiology, and clinical history, must be interpreted in concert [34–37]. To our knowledge, this study is among the first to apply a frontier LLM to support multidisciplinary treatment planning in pancreatic cancer, using real-world data and expert consensus as ground truth. It thus extends the existing literature by demonstrating that adv. prompting strategies can unlock meaningful clinical reasoning capabilities from LLMs in complex and decision-critical contexts.

Future research should focus on expanding dataset size and diversity to encompass broader clinical scenarios and patient populations. Integrating additional data modalities, such as structured laboratory results, molecular profiling, and imaging pixel data, could further refine model accuracy and clinical applicability. Moreover, exploring fine-tuning or domain-adaptive training techniques may enhance the model’s capacity to grasp domain-specific nuances and rare clinical presentations. Prospective clinical trials will be pivotal to rigorously evaluate the safety, effectiveness, and usability of LLM-assisted tumor board decision support systems, ensuring that such tools complement human expertise while maintaining patient safety and ethical standards.

In summary, the principal finding of this study is the instability and clinical insufficiency of global accuracy metrics in an imbalanced, high-stakes oncologic setting. While the model effectively approximates majority-class decisions (SURG and PALL), it systematically fails in clinically nuanced, intermediate-stage cases requiring neoadjuvant pathways. High overall accuracy can thus mask clinically significant blind spots, demonstrating that current LLMs do not yet possess the reliability required for expert-level approximation in tumor boards.

## Conclusion

Large language models like LLaMA 3.3 (70b) can approximate majority-class tumor board decisions (surgical and palliative) for pancreatic cancer, but they exhibit a critical systematic failure in identifying complex, intermediate-stage candidates for neoadjuvant therapy. The principal finding of this study is that high global accuracy can mask clinically significant blind spots in therapeutically critical minority classes. Consequently, while prompt engineering alters error distribution, current models lack the reliability and nuanced clinical reasoning required for expert-level approximation. They are not ready for clinical consideration without rigorous oversight and specific architectural adaptations to handle severe class imbalance and clinical ambiguity.

## Supplementary Information

Below is the link to the electronic supplementary material.

Supplementary Material 1

## Data Availability

The datasets generated and/or analysed during the current study are not publicly available due to patient data protection and institutional regulations but are available from the corresponding author on reasonable request.

## Abbreviations

AI: Artificial Intelligence
adv.: advanced (prompting strategy)
CI: Confidence Interval
CoT: Chain-of-Thought
CT: Computed Tomography
ERCP: Endoscopic Retrograde Cholangiopancreatography
IRB: Institutional Review Board
LAPC: Locally Advanced Pancreatic Cancer
LLM: Large Language Model
MRI: Magnetic Resonance Imaging
NCCN: National Comprehensive Cancer Network
NEO: Neoadjuvant Therapy / Chemotherapy
PALL: Palliative Therapy
PDAC: Pancreatic Ductal Adenocarcinoma
SMA: Superior Mesenteric Artery (A. mesenterica sup.)
SMV: Superior Mesenteric Vein (V. mesenterica sup.)
SURG: Surgical Resection
