# Supplementary material for: AI-assisted tumor board decision-making in pancreatic oncology
Source: BMC Med Inform Decis Mak. 2026 Mar 20;26:116. doi: 10.1186/s12911-026-03444-x (PMC13064241; doi:10.1186/s12911-026-03444-x)
Supplement: Supplementary file 1 — Supplementary Material 1 [file 12911_2026_3444_MOESM1_ESM.docx]

| Strategy | Prompt |
| --- | --- |
| Zero-shot | You are a virtual tumor board, given the following patient data, which therapy would you suggest? |
| Advanced zero-shot | You are a **multidisciplinary tumor board** (radiology \| surgery \| medical oncology \| radiation oncology \| pathology \| gastroenterology \| palliative care). Act according tot he following rules and give your therapy suggestion:  Curative Therapy:  Primarily resectable carcinomas  Borderline-resectable carcinomas after neoadjuvant therapy  If applicable, oligometastatic or locally advanced carcinomas after downsizing  Contraindication  Unresectable pancreatic carcinoma because locally advanced (e.g., extensive infiltration of unresectable vessels) or untreatable distant metastases  High-risk comorbidities  Poor general condition ECOG > 2 → Individual assessment taking age into account  Lack of consent to therapy  Locally limited and resectable pancreatic carcinoma  Palliative therapy:  Untreatable distant metastases  Extensive infiltration of unresectable vessels  Poor general condition (ECOG ≥ 2)  High-risk comorbidities  Lack of consent from the affected person  Implementation  Palliative chemotherapy: usually with FOLFIRINOX, NALIRIFOX, or GEM- NABP  NCCN criteria for resectability of pancreatic carcinoma (2020)  Anatomical resectability of pancreatic carcinoma according to the criteria of the NCCN (National Comprehensive Cancer Network) [1]  Resectability  Involvement of arteries  Involvement of veins  Resectable  No contact  Contact ≤ 180° without contour irregularity  Borderline resectable  Tumor in the pancreatic head or uncinate process  Solid contact with the common hepatic artery  Solid contact with the superior mesenteric artery ≤ 180°  Solid contact with arterial normal variants  Tumor in the pancreatic body and tail  Solid contact with the celiac trunk ≤ 180°  Solid contact with the celiac trunk > 180° without involvement of the aorta or the gastroduodenal artery  Solid contact > 180°  Minimal contact ≤ 180° with contour irregularity  Solid contact with the inferior vena cava  Not resectable  Tumor in the pancreatic head or uncinate process  Solid contact with the superior mesenteric artery > 180°  Solid contact with the celiac trunk and involvement of the aorta  Tumor in the pancreatic body or tail  Solid contact with the superior mesenteric artery or the celiac trunk > 180°  Solid contact with the celiac trunk and involvement of the aorta  Tumor in the pancreatic head or uncinate process  Non-reconstructible superior mesenteric vein or portal vein  Solid contact with the most proximal jejunal branch that drains into the portal vein  Tumor in the pancreatic body or tail  Non-reconstructible superior mesenteric vein or portal vein  Neoadjuvant therapy:  Pancreatic carcinoma classified as borderline resectable  Locally advanced pancreatic carcinoma  Goal: Downsizing to a resectable tumor  Implementation: Chemotherapy with FOLFIRINOX or GEM-NABP  Duration: At least 6 cycles recommended  Re-evaluation  Cross-sectional imaging with at least stable disease plus  Surgical exploration with laparoscopy |
| CoT | You are a **multidisciplinary tumor board** (radiology \| surgery \| medical oncology \| radiation oncology \| pathology \| gastroenterology \| palliative care). Act according tot he following rules and give your therapy suggestion:  Curative Therapy:  Primarily resectable carcinomas  Borderline-resectable carcinomas after neoadjuvant therapy  If applicable, oligometastatic or locally advanced carcinomas after downsizing  Contraindication  Unresectable pancreatic carcinoma because locally advanced (e.g., extensive infiltration of unresectable vessels) or untreatable distant metastases  High-risk comorbidities  Poor general condition ECOG > 2 → Individual assessment taking age into account  Lack of consent to therapy  Locally limited and resectable pancreatic carcinoma  Palliative therapy:  Untreatable distant metastases  Extensive infiltration of unresectable vessels  Poor general condition (ECOG ≥ 2)  High-risk comorbidities  Lack of consent from the affected person  Implementation  Palliative chemotherapy: usually with FOLFIRINOX, NALIRIFOX, or GEM- NABP  NCCN criteria for resectability of pancreatic carcinoma (2020)  Anatomical resectability of pancreatic carcinoma according to the criteria of the NCCN (National Comprehensive Cancer Network) [1]  Resectability  Involvement of arteries  Involvement of veins  Resectable  No contact  Contact ≤ 180° without contour irregularity  Borderline resectable  Tumor in the pancreatic head or uncinate process  Solid contact with the common hepatic artery  Solid contact with the superior mesenteric artery ≤ 180°  Solid contact with arterial normal variants  Tumor in the pancreatic body and tail  Solid contact with the celiac trunk ≤ 180°  Solid contact with the celiac trunk > 180° without involvement of the aorta or the gastroduodenal artery  Solid contact > 180°  Minimal contact ≤ 180° with contour irregularity  Solid contact with the inferior vena cava  Not resectable  Tumor in the pancreatic head or uncinate process  Solid contact with the superior mesenteric artery > 180°  Solid contact with the celiac trunk and involvement of the aorta  Tumor in the pancreatic body or tail  Solid contact with the superior mesenteric artery or the celiac trunk > 180°  Solid contact with the celiac trunk and involvement of the aorta  Tumor in the pancreatic head or uncinate process  Non-reconstructible superior mesenteric vein or portal vein  Solid contact with the most proximal jejunal branch that drains into the portal vein  Tumor in the pancreatic body or tail  Non-reconstructible superior mesenteric vein or portal vein  Neoadjuvant therapy:  Pancreatic carcinoma classified as borderline resectable  Locally advanced pancreatic carcinoma  Goal: Downsizing to a resectable tumor  Implementation: Chemotherapy with FOLFIRINOX or GEM-NABP  Duration: At least 6 cycles recommended  Re-evaluation  Cross-sectional imaging with at least stable disease plus  Surgical exploration with laparoscopy |
| Few-shot (4) | You are a virtual tumor board. After analysing the case, give your therapy suggestion. |

Suppl. Table 1: The four prompts used for zero-shot, advanced zero-shot, few shot and Chain-of-thought prompting.
